# Multiple-Color Optical Activation, Silencing, and Desynchronization of Neural Activity, with Single-Spike Temporal Resolution

**DOI:** 10.1371/journal.pone.0000299

**Published:** 2007-03-21

**Authors:** Xue Han, Edward S. Boyden

**Affiliations:** 1 Stanford University School of Medicine, Stanford, California, United States of America; 2 Massachusetts Institute of Technology Media Lab, Massachusetts Institute of Technology, Cambridge, Massachusetts, United States of America; University of Minnesota, United States of America

## Abstract

The quest to determine how precise neural activity patterns mediate computation, behavior, and pathology would be greatly aided by a set of tools for reliably activating and inactivating genetically targeted neurons, in a temporally precise and rapidly reversible fashion. Having earlier adapted a light-activated cation channel, channelrhodopsin-2 (ChR2), for allowing neurons to be stimulated by blue light, we searched for a complementary tool that would enable optical neuronal inhibition, driven by light of a second color. Here we report that targeting the codon-optimized form of the light-driven chloride pump halorhodopsin from the archaebacterium *Natronomas pharaonis* (hereafter abbreviated Halo) to genetically-specified neurons enables them to be silenced reliably, and reversibly, by millisecond-timescale pulses of yellow light. We show that trains of yellow and blue light pulses can drive high-fidelity sequences of hyperpolarizations and depolarizations in neurons simultaneously expressing yellow light-driven Halo and blue light-driven ChR2, allowing for the first time manipulations of neural synchrony without perturbation of other parameters such as spiking rates. The Halo/ChR2 system thus constitutes a powerful toolbox for multichannel photoinhibition and photostimulation of virally or transgenically targeted neural circuits without need for exogenous chemicals, enabling systematic analysis and engineering of the brain, and quantitative bioengineering of excitable cells.

## Introduction

Much effort in neuroscience is directed towards determining how neural activity in a specific set of neurons contributes to a particular neural computation, behavior, or pathological state. Over the last hundred years, thousands of studies have investigated the neural substrates necessary and sufficient for sensation, perception, cognition, and action, via pharmacological or ablative neural silencing (or studies of human patients with localized lesions), or localized electrical microstimulation of specified neural circuits (e.g., [1]–[11]). Technologies for targeted neural silencing or stimulation have also found clinical uses for treatment of many neurological and psychiatric disorders including Parkinson's disease, cluster headache, drug-resistant depression, and a variety of other hard-to-treat disorders (e.g., [12]–[17]). The use of neural control technologies in these two complementary quests–the analysis, and engineering, of the brain–would be greatly aided by a tool for reliably activating and inactivating genetically-targeted neurons in a temporally-precise and rapidly-reversible fashion. A toolbox for bi-directionally sculpting activity in targeted cell types at the millisecond timescale could greatly help answer a number of outstanding classical questions in neuroscience, and enable completely new and previously-intractable vistas to be explored: What are the precise brain regions, cell types, and neural activity patterns required at each phase–sensory, decision-making, motor–of a specific behavior? To mediate a particular perception, feeling, decision, memory, or action, what is the precise number of neurons that must be active within a certain region, and when and how long must they be active? What is the causal role of neural synchrony and precise spike timing in neural computation, plasticity, and circuit pathology? As memories are encoded, consolidated, actively reversed, and forgotten, how do the critical neural loci of memory change? How can we exert control over the neural circuits left in disarray by a myriad of neurological and psychiatric diseases, and correct the activity patterns so as to restore normal brain function?

Having previously adapted a light-activated cation channel from green algae, channelrhodopsin-2 (ChR2), for sensitizing targeted neurons to temporally-precise excitation by pulses of blue light [18], we searched for a complementary optical tool that would enable simultaneously-and independently-controllable neuronal inhibition, driven by light of a second color. In recent years, a diversity of methods have been proposed for reversibly inactivating neurons [19]–[26]; the majority of these methods rely upon chemical delivery and washout to temporally delimit the duration of inactivation, which accordingly limits the induction and reversal kinetics to periods of at least several minutes or hours. One tantalizing study has shown that expressing the rat protein rhodopsin 4 (RO4) in neurons can enable light-driven G-protein-mediated neural inhibition [27]. Perhaps due to the multiple-component G-protein signaling pathway involved, induction of inhibition was partial, and proceeded with a time constant of ∼300 ms, with release from inhibition proceeding with a time constant of ∼6 seconds after light shutoff. In contrast, optical activation via ChR2 offers neural excitation with temporal resolution on the order of 1 millisecond; the ability to optically silence neurons on the same timescale, and especially to exert bidirectional control of membrane voltage with two colors of light, would clearly be of enormous benefit both as a basic science tool and as a novel bioengineering building-block.

We screened single-component, type I microbial opsins with significant homology to ChR2, which has been shown to possess these important properties in the context of optical activation of mammalian neurons [18], [28], [29]. Specifically, we surveyed the class of archaebacterial light-activated chloride pumps called halorhodopsins [30]–[33], which are seven-transmembrane proteins capable of driving inward chloride currents in response to green-yellow light (525–650 nm). The archaebacteria that express halorhodopsins typically live in environments with very high chloride concentrations (e.g., 1–5 M), and use halorhodopsin to pump chloride into the cell (even against the membrane potential) in order to maintain osmotic balance and accordingly reduce energy expenditure. Some wild-type halorhodopsins have been shown to preserve functionality at much lower chloride concentrations, comparable to those found in mammalian cerebrospinal fluid [30], [34]. Here we report that targeting the mammalian codon-optimized form of halorhodopsin from the archaebacterium *Natronomas pharaonis* (hereafter abbreviated Halo) to genetically-specified neuron subtypes enables them to be silenced reliably and reversibly by millisecond-timescale pulses of yellow light. We demonstrate that rapidly-alternating pulses of yellow and blue light can drive high-fidelity, naturalistic trains of neural hyperpolarizations and depolarizations in neurons simultaneously expressing yellow light-driven Halo and blue light-driven ChR2, enabling for the first time experiments that perturb neural synchrony without alteration of spike rate. In all of these experiments, no chemical supplementation was needed to enable Halo or ChR2 function in mammalian neurons. Thus, the ChR2/Halo system constitutes a powerful toolbox enabling multichannel photoinhibition and photostimulation of virally-or transgenically-targeted neural circuits, enabling the systematic analysis and engineering of neural circuits, as well as the quantitative bioengineering of excitable cells.

## Results

**Figure 1:**
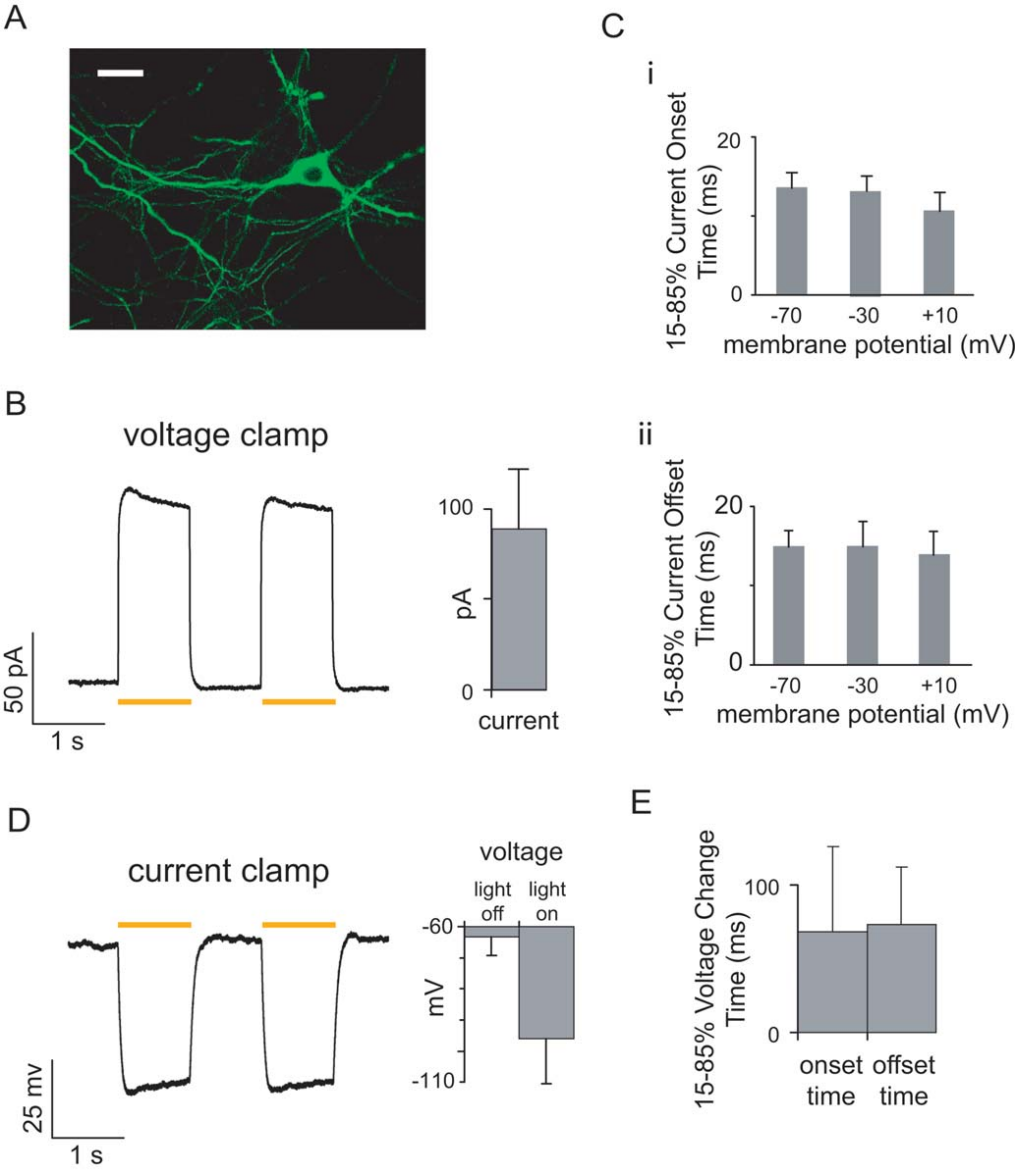
Millisecond-timescale Halo-mediated neuronal hyperpolarization, elicited by pulses of yellow light. (A) A representative cultured hippocampal neuron expressing mammalian codon-optimized *N. pharaonis* halorhodopsin (abbreviated Halo) fused to GFP, under the CaMKII promoter. Scale bar, 20 µm. (B) Neuronal currents elicited by optical activation of Halo. *Left*, representative outward currents elicited by two 1-second pulses of yellow (560±27.5 nm) light (∼10 mW/mm^2^) in a voltage-clamped neuron held at −70mV. *Right*, population data for *n* = 22 neurons. In this and subsequent figures, gray bars represent mean ± standard deviation unless otherwise indicated. Yellow bars in this and subsequent figures represent the period of yellow light exposure. (C) Kinetic properties of yellow light-elicited, Halo-mediated currents from voltage-clamped neurons. (i), 15–85% current onset time; (ii), 85–15% offset time. For each measurement, data is presented from neurons held at −70 mV, −30 mV and+10 mV (*left* to *right*). In this panel, gray bars represent mean ± standard error of the mean (S.E.M.). (D) Neuronal hyperpolarizations elicited by optical activation of Halo. *Left*, representative membrane voltage hyperpolarizations elicited by two 1-second pulses of yellow light, in a current-clamped neuron held at resting membrane potential. *Right*, population data for *n* = 19 neurons. (E) Kinetic properties of yellow light-elicited, Halo-mediated hyperpolarizations from current-clamped neurons, including both 15–85% voltage change onset time and 85–15% offset time.

We first created a fusion protein comprising the mammalian codon-optimized form of *N. pharaonis* halorhodopsin (Halo), with EGFP added in-frame at the C-terminus for ease of visualization (see Methods for details). When expressed in cultured hippocampal neurons using the CaMKII promoter, which targets excitatory neurons of the forebrain [35], Halo-EGFP fluoresced brightly, and appeared evenly distributed around the neuron (Fig. 1A). When exposed to ∼10 mW/mm^2^ yellow light (from a xenon lamp, filtered by a standard Texas red excitation filter from Chroma, bandpass 560±27.5 nm), voltage-clamped hippocampal neurons expressing Halo experienced outward currents with rapid onset, stable steady-state amplitude, and abrupt shut-off after cessation of illumination. No supplementation of culture or recording media with the essential halorhodopsin cofactor all-trans retinal was necessary for strong currents to be elicited, consistent with prior work that demonstrated high enough levels of all-trans retinal in mammalian culture and brain to enable type I opsin functionality [18], [28]. Light pulses elicited pulse amplitudes of 88.7±32.8 pA (mean±st. dev.; *n* = 22 neurons; Fig. 1B). Repeating a 1-second pulse of yellow light twice, spaced by 1 second of darkness, resulted in similar pulse amplitudes each time (*p*>0.50, paired t-test), although during each light pulse, a slight perceptible decay was visible (analyzed later in the manuscript). This relatively stable current amplitude is consistent with what is known about the halorhodopsin photocycle, which can fully complete within tens of milliseconds [33]. The light-elicited current amplitude did not vary significantly with holding voltage when assayed at-70 mV, −30 mV, and+10 mV (F = 0.004, *p*>0.95, ANOVA with factor of holding voltage), nor did any measured kinetic parameters vary across this voltage range, such as onset or offset times of current pulses (F<0.6, *p*>0.55 for all comparisons, ANOVA; Fig. 1C). The onset and offset times of elicited currents were strikingly rapid, ∼10–15 ms at all holding voltages tested (Fig. 1Ci, 1Cii). When held in current clamp, hippocampal neurons underwent peak hyperpolarizations of 32.9±14.4 mV (*n* = 19 neurons) in response to pulses of yellow light, with no difference between the peak hyperpolarizations achieved by two 1-second pulses separated by a 1-second pause (*p*>0.85, paired t-test; Fig. 1D). Furthermore, as expected from the current-clamp experiments, these large voltage changes were quite rapid, with onset and offset times of 68±57 and 73±39 ms respectively for these large voltage swings (Fig. 1E). Thus, Halo was capable of reliably mediating hyperpolarizations of large magnitude, with fast onset and offset times at the beginning and end of light exposure.

**Figure 2:**
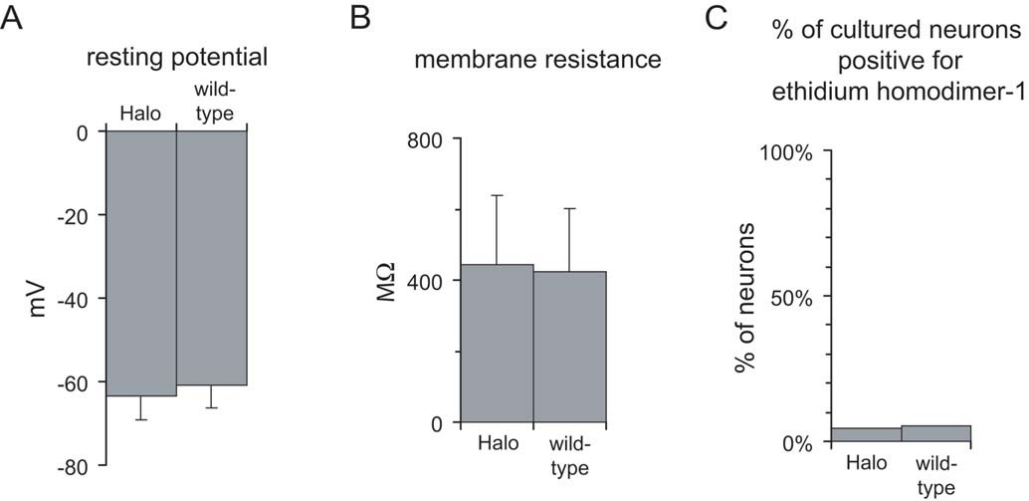
Safety of Halo in cultured hippocampal neurons. (A) Membrane resting potential of Halo-expressing vs. wild-type neurons (*n* = 19 each). (B) Membrane resistance of Halo-expressing vs. wild-type neurons. (C) Fraction of Halo-expressing (*n* = 22) vs. wild-type (*n* = 308) cultured neurons whose nuclei stained positive for the membrane-integrity assessing DNA stain ethidium homodimer-1.

Since it is important to evaluate whether a new technology has unanticipated side effects, such as altering basal cell physiology, or increasing the propensity for cell death, we conducted several control experiments. First, we characterized the basal state of Halo-expressing neurons electrophysiologically when no light was present. When measured in darkness, there was no difference in the resting potentials of neurons expressing Halo, and those of neighboring wild-type neurons (*p*>0.20, t-test; *n* = 19 Halo-expressing cells, *n* = 19 wild-type cells; Fig. 2A). Similarly, membrane resistance was not significantly different between the Halo-expressing cells and the wild-type cells (*p*>0.70; Fig. 2B). This result suggests that basal neural activity would be little affected by the presence of Halo in the absence of light. As an independent assay for unanticipated effects on cell health, we assayed whether Halo expression for one week in cultured hippocampal neurons could lead to apoptosis, using the membrane-impermeant DNA stain ethidium homodimer-1 to detect any cell membrane breakdown that would accompany apoptotic cell death [36]. We found no difference in cell death between Halo-expressing and control wild-type neurons: 16/308 (5.2%) control neurons counted, and 1/22 (4.5%) Halo-expressing neurons counted, were labeled by ethidium homodimer-1, indicating that Halo was not toxic over the timecourse of a week of expression (χ^2^ = 0.02, *p*>0.85; Fig. 2C). Thus, along multiple axes, Halo proved to be well-tolerated by mammalian neurons, perhaps as expected given its structural similarity to the well-tolerated photostimulation protein Channelrhodopsin-2 [18], [28], [29].

**Figure 3:**
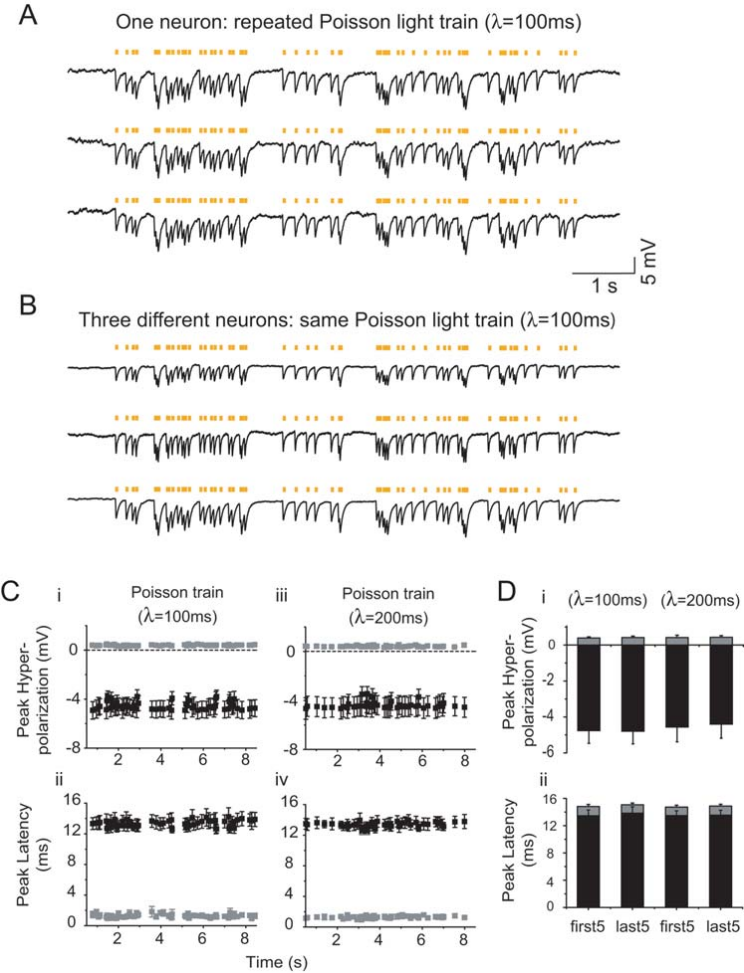
Halo-mediated naturalistic trains of inhibitory events. (A) Three voltage traces of a representative current-clamped hippocampal neuron, exposed to a Poisson train of yellow light pulses. Each light pulse lasts 10 ms, and the Poisson train has a mean inter-pulse interval of λ = 100 ms. (B) Voltage traces of three different representative current-clamped neurons exposed to the same Poisson train of light pulses (λ = 100 ms). (C) Properties of hyperpolarization events elicited by Poisson trains with inter-pulse interval λ = 100 ms (i, ii) and λ = 200 ms (iii, iv), plotted versus onset time of each light pulse. Plots (i) and (iii) show the peak amplitude of each hyperpolarization event (black symbols), as well as the across-trials standard deviation of these amplitude values across ten trials (gray symbols). Plots (ii) and (iv) show the latency between the onset time of the light pulse and the time of the hyperpolarization peak (black symbols), as well as the across-trials standard deviation of these timing values across ten trials (gray symbols). In this panel, plotted points are across-neuron mean±S.E.M. (*n* = 5 neurons). (D) Comparison of the peak hyperpolarization (i) and the time-to-peak (ii) data between the beginning (first5) and end (last5) of each Poisson train, for the *n* = 5 neurons described in Fig. 3C. In (i): for each neuron, the average of the first 5 or last 5 hyperpolarization peaks (black) or the across-trials standard deviation of these amplitude values (gray) was first computed, then the across-neuron mean±S.E.M. was plotted. In (ii): for each neuron, the average of the first 5 or last 5 times-to-peak (black) or the across-trials standard deviation of these times-to-peak (gray) were first computed, then the across-neuron mean±S.E.M. was plotted. For (ii), the gray bars were stacked on top of the black ones for ease of visualization.

We next probed whether the fast response times of Halo could support naturalistic sequences of hyperpolarization events, in response to trains of brief pulses of yellow light. Figure 3A shows three traces of hyperpolarization events elicited in a single neuron, resulting from repeatedly playing back a Poisson train (mean inter-pulse interval, λ = 100 ms, 59 pulses), of 10 ms-duration yellow light pulses, to illustratively simulate stochastic inhibitory (e.g., GABAergic) synaptic input. Figure 3B shows three such hyperpolarization traces, taken from different neurons. The variability of such trains was remarkably low in many regards–across ten repeated trials in a single cell, across multiple cells (*n* = 5 neurons), and over time throughout a sustained train of 59 pulses (Fig. 3C, 3D). Notably, we found that for hyperpolarizations elicited by 10 ms-duration light pulses during a λ = 100 ms Poisson train, the mean amplitude was −4.56 mV (averaged across trials and neurons), but the trial-to-trial standard deviation of this amplitude was only 400 µV (averaged across neurons, Fig 3Ci and Fig. 3Di, left side). The trial-to-trial jitter of the time the hyperpolarization took to reach its peak value was also small, 1.27 ms (averaged across neurons, Fig. 3Cii and Fig. 3Dii, left side). The neuron-to-neuron variability of amplitude and timing was somewhat larger than the trial-to-trial variability, with standard deviations of 1.45 mV and 1.78 ms, respectively, but these values nevertheless demonstrated that precise inhibitory control of a population of neurons could take place with millivolt and millisecond resolution. Finally, we quantitatively examined the through-train sustainability of light-elicited voltage changes, by comparing the amplitude mean and amplitude variability, and timing variability, between the hyperpolarization events elicited by the first five light pulses in the 59-pulse train, and the last five light pulses in the train (Figs. 3Di and 3Dii, left side). No difference was seen for any of these statistics, compared between the events elicited at the beginning vs. end of the train (*p*>0.10 for all measures, t-test). Identical conclusions held for the λ = 200 ms Poisson train with 46 pulses (Figs. 3Ciii and 3Civ, and Figs. 3Di and 3Dii, right side). The high temporal and amplitude fidelity of Halo-mediated hyperpolarizations suggests that Halo may be an ideal tool for simulating many forms of synaptic inhibition.

**Figure 4:**
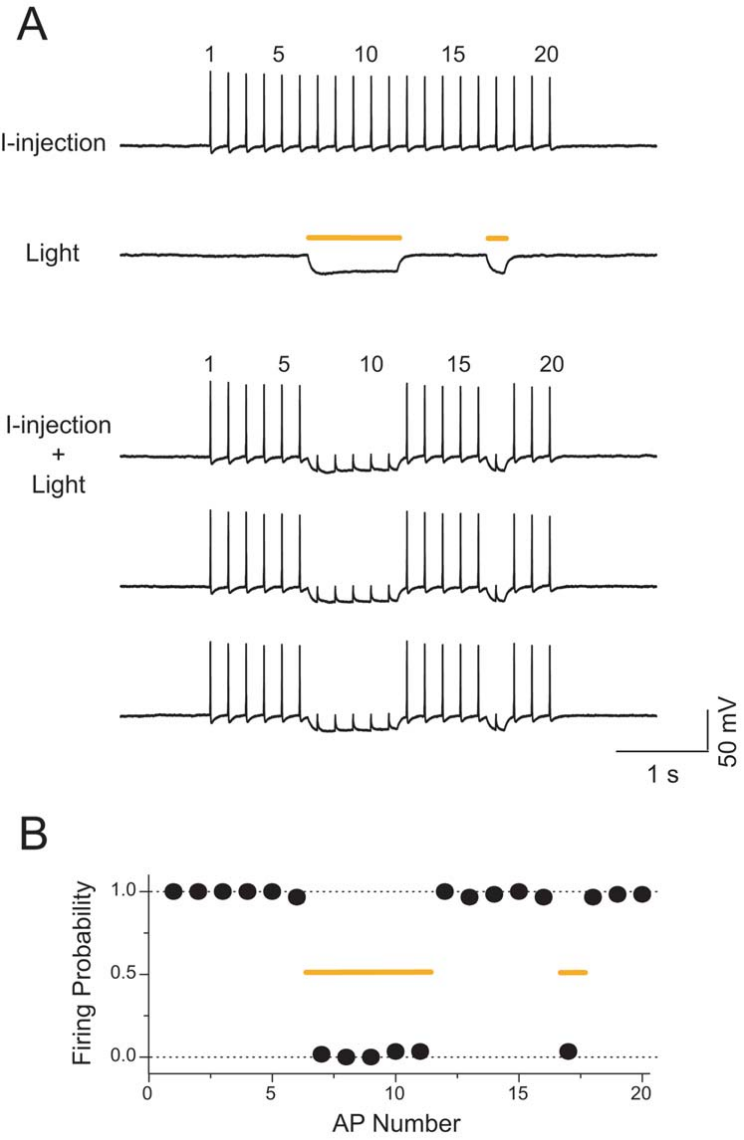
Halo-mediated silencing of neuronal spiking. (A) Light-driven spike blockade, demonstrated for a representative hippocampal neuron. *Top* (“I-injection”), neuronal firing of 20 spikes at 5 Hz, induced by pulsed somatic current injection (∼300 pA, 4 ms). *Middle* (“Light”), membrane hyperpolarization induced by two periods of yellow light, timed so as to be capable of blocking spikes 7–11 and spike 17, out of the train of 20 spikes. *Bottom* (“I-injection±Light”), yellow light drives Halo to block neuron spiking (note absence of spikes 7–11 and of spike 17), while leaving spikes elicited during periods of darkness largely intact. (B) Population data (*n* = 6 neurons) for light-driven, Halo-mediated spike blockade, showing high spike probability during periods of darkness (spikes 1–6, 12–16, and 18–20), and low spike probability during periods of yellow light illumination (spikes 7–11 and spike 17). Error bars (S.E.M.) are smaller than the points plotted.

We next analyzed the ability of Halo to enable rapidly-inducible and rapidly-reversible silencing of neural activity. In patch clamped neurons, we delivered trains of intracellularly-injected somatic currents (∼300 pA, lasting 4 ms each), causing the neurons to fire 20 action potentials at 5 Hz with a 100% success rate (Fig. 4A, “I-injection”). We then scheduled yellow light pulses to occur at certain phases within the somatic current-injection protocol–specifically, during the times when spikes 7 through 11, and spike 17, would normally be driven (Fig. 4A, “Light”). Finally, we presented the light pulses and the somatic current injections simultaneously (Fig. 4A, “I-injection±light”, three trials shown), and discovered that strikingly, spiking was blocked precisely during the periods of yellow light exposure, and at no other times. Most remarkably, the rapid onset and offset kinetics of Halo allowed the *deletion of even single spikes*–specifically, the second yellow light pulse, timed to silence just spike 17, was able to eliminate spike 17 without affecting neighboring spikes 16 or 18 at all. We repeated this experiment five times, on each of *n* = 6 neurons (Fig. 4B). Across all these experiments, the second pulse of yellow light reduced the probability of firing spike 17 to 3.3%, whereas neighboring spikes 16 and 18 fired 96.7% of the time; this latter success probability was not significantly different from the success rate of the first spike in the train, before any light exposure at all (χ^2^ = 1.02, *p*>0.30). In total, during periods when the yellow light was off, somatic current pulses elicited spikes 98.7% of the time, whereas during periods when the yellow light was on, somatic current pulses elicited spikes only 1.2% of the time. The temporal precision of Halo in silencing spikes therefore offers a novel method of creating ultratransient, precise, and effective inhibition of activity in genetically-specified neurons.

**Figure 5:**
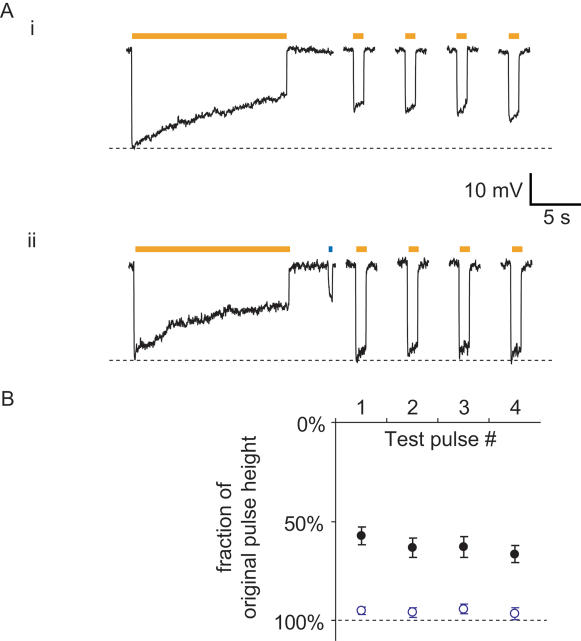
Blue light facilitates optimal Halo function. (A) (i) Timecourse of Halo-mediated hyperpolarizations in a representative current-clamped hippocampal neuron during 15 seconds of continuous yellow light, followed by four 1-second test pulses of yellow light (one every 30 seconds, starting 10 seconds after the end of the first 15-second period of yellow light). (ii) Timecourse of Halo-mediated hyperpolarization for the same cell exhibited in (i), but when Halo function is facilitated by a 400-ms pulse of blue light in between the 15-second period of yellow light and the first 1-second test pulse. (B) Population data for blue-light facilitation of Halo recovery (*n* = 8 neurons). Plotted are the hyperpolarizations elicited by the four 1-second test pulses of yellow light, normalized to the peak hyperpolarization induced by the original 15-second yellow light pulse. Dots represent mean±S.E.M. Black dots represent experiments when no blue light pulse was delivered (as in Fig. 5Ai.). Open blue dots represent experiments when 400 ms of blue light was delivered to facilitate recovery (as in Fig. 5Aii.).

Halo-mediated currents and hyperpolarizations appeared to decay slightly during 1-second long pulses of yellow light (Fig. 1B, D), raising the question of how Halo might perform during longer-duration neural silencing experiments. We exposed Halo-expressing neurons to continuous yellow light, and found that the hyperpolarization decayed with a time constant of approximately 16.8±10.4 seconds (*n* = 8 neurons; representative trace shown in Fig. 5Ai). We tested for recovery of Halo function after 15 seconds of yellow light, with repeated 1-second test pulses of yellow light delivered every 30 seconds, and found that even after four such rest periods, the hyperpolarization peak amplitude remained down by 33% from its original peak amplitude, suggesting that the Halo protein had entered an inactive state (Fig. 5Ai and Fig. 5B, black dots). Earlier studies on halorhodopsin from another archaebacterium, *Halobacterium halobium*, found evidence for a similar type of rundown, due to accumulation of an inactive 13-*cis* retinal form of the halorhodopsin molecule, which required seconds to recover to the original state [37]. However, these earlier studies also found that brief periods of blue light could facilitate rapid recovery of *H. halobium* halorhodopsin to the active form, by assisting in the re-isomerization of 13-cis retinal to the all-trans form. For Halo, we found that a brief pulse of moderate-intensity blue light (∼10 mW/mm^2^ 400 ms-long), delivered through the GFP excitation filter) could completely restore Halo to its active state (Fig. 5Aii), recovering the hyperpolarizations elicited by the test pulses of yellow light to their original amplitude (*p*>0.05 for each test pulse, paired t-test; *n* = 8; Fig. 5B, open blue dots). Thus, despite the existence of rundown during long-lasting exposure of Halo-expressing neurons to yellow light, brief periods of blue light exposure will facilitate the optimal performance of Halo during long periods of neural inhibition *in vivo*.

**Figure 6:**
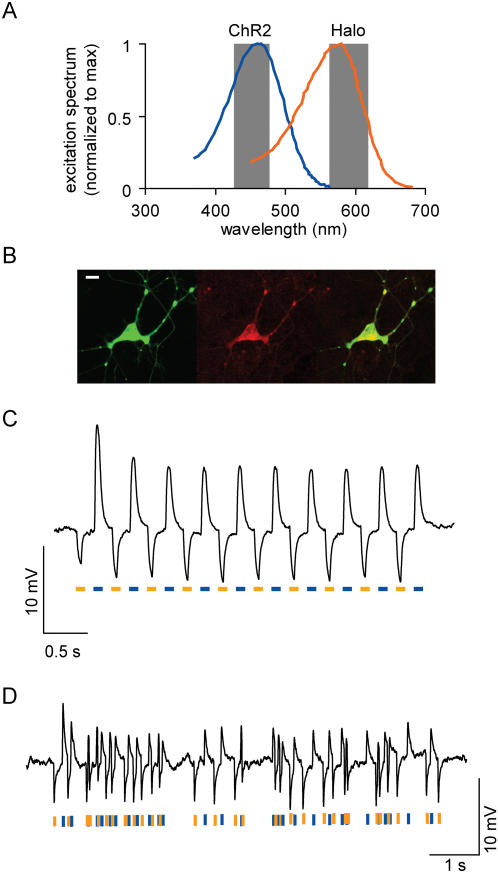
Bi-directional optical control of voltage with blue and yellow light pulses. (A) Action spectrum for ChR2 (blue, adapted from [71]), overlaid with absorption spectrum for *N. pharaonis* halorhodopsin (orange, adapted from [32]). Each spectrum is normalized to its own peak, for ease of comparison. Gray bars denote bandpass windows of commercially-available Chroma HQ450/50× and HQ590/55× filters, which may be useful for high-fidelity fast-wavelength switching between ChR2 photostimulation and Halo photoinhibition. (B) Co-expression of ChR2-mCherry (*left*) and Halo-GFP (*middle*) in a single representative cell expressing both (*right*, overlay). Scale bar, 20 µm. (C) Hyperpolarization and depolarization events elicited in a single representative neuron, by two interleaved 2.5 Hz trains of yellow and blue light pulses (50 ms duration), denoted by bars of respective coloration. (D) Hyperpolarization and depolarization events induced in a representative neuron by a Poisson train (mean inter-pulse interval λ = 100 ms) of alternating pulses of yellow and blue light (10 ms duration), denoted by appropriate colors.

We recently showed that one member of the type I opsin family, the light-activated cation channel Channelrhodopsin-2 (ChR2), could mediate neural excitation in response to pulses of blue light (∼470 nm, delivered through a standard GFP excitation filter) [18]. Furthermore, the excitation peak of ChR2 is spectrally separated far enough from Halo that commonly-available fluorescence excitation filters (e.g., HQ450/50x and HQ590/55×) may be sufficient to enable neural photostimulation and photoinhibition (illustrated in Fig. 6A). We targeted individual neurons simultaneously with both Halo and the human codon-optimized form of ChR2 (with the red fluorophore mCherry [38] fused to the C-terminus, for ease of visualization), both under control of the CaMKII promoter (Fig. 6B). mCherry was visualized with the same Texas red filter used to stimulate Halo (see [38] for detailed spectral properties). Note that due to spectral overlap, mCherry visualization (with yellow light) can result in Halo stimulation, and GFP visualization (with blue light) can result in ChR2 stimulation, so from a practical standpoint using the ChR2/Halo system as here configured requires cell identification and voltage control to occur during distinct experimental stages. We found that such neurons could respond to rapidly-switched pulses of yellow and blue light with hyperpolarizations and depolarizations respectively (Fig. 6C). Poisson trains (mean inter-pulse interval λ = 100 ms) of rapidly-alternating yellow and blue light pulses elicited rapidly-alternating hyperpolarizations and depolarizations in the same neuron (Fig. 6D). The ability to drive excitation and inhibition in the same neuron, using two different wavelengths of light, may open up the ability to answer questions for which no current technology permits resolution.

**Figure 7:**
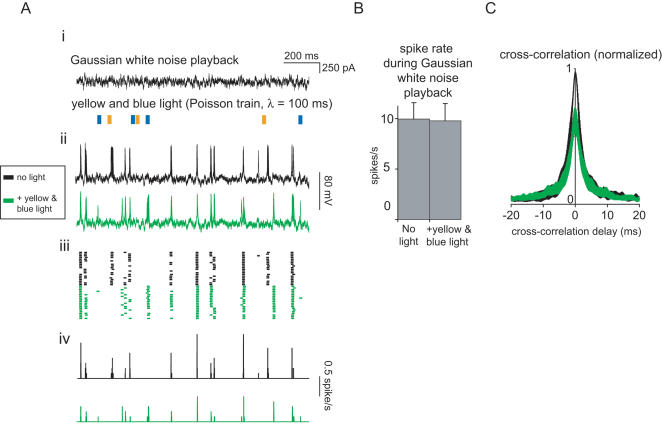
Multichannel optical disruption of precise spike timing, without alteration of spike rate. (A) Optical disruption of spike timing, without alteration of spike rate, for a representative neuron expressing both ChR2 and Halo. (i), stimulus traces showing subsegments of the somatically injected filtered Gaussian white noise current used in all these experiments (top), as well as of the Poisson train (mean inter-pulse interval λ = 100 ms) of alternating yellow and blue light pulses (bottom). (ii), twenty-trace overlays of voltage responses to the somatically injected white noise current, either with no light (top, black traces) or with delivery of a Poisson train of yellow and blue light pulses (bottom, green traces). (iii), spike raster plots for the traces shown in Fig. 7Aii. (iv), spike-timing histograms (bin size: 500 µs) for the rasters shown in Fig. 7Aiii. (B) Spike rates of neurons (*n* = 7) injected with filtered Gaussian white noise current, either with no light (left) or with concurrent delivery of a Poisson train of yellow and blue light pulses (right). Plotted is mean±S.E.M. (C) Cross-correlation between spike trains elicited from the same filtered Gaussian white noise current injection, played twice, when either both current injections were performed in the dark (black curve), or when one of the current injections was performed with concurrent delivery of a Poisson train of yellow and blue light pulses (green trace). Data is plotted as mean±S.E.M (averaged across *n* = 7 neurons). See Methods for details.

One prominent example of such a long-standing question is whether synchrony or precise timing of neural activity has an important or causal role in neural computation. Such activity has been observed during, or associated with, various brain functions such as timing-dependent plasticity, global stimulus feature processing, visuomotor integration, motor planning, and attention (e.g., [39]–[43]), and abnormal patterns of neural synchrony have been associated with a variety of neurological and psychiatric disorders [44]. To date, however, no generalized strategy has permitted the disruption of neural synchrony or precisely-timed spiking without the confound of altering spike rate. To examine whether Halo and ChR2 together could enable this kind of experiment, we generated precisely-timed spike trains by repeatedly injecting cultured hippocampal neurons with a single filtered Gaussian white noise current trace (see Fig. 7Ai, top, for a fragment thereof; see Methods for details). Such noisy currents had been previously found to induce reliable spike trains in current-clamped neurons embedded in cortical brain slices [45]. On some trials, we concurrently illuminated the neuron with the Poisson train of alternating yellow and blue light pulses shown in Fig. 6D (see Fig. 7Ai, bottom). We found that when delivered alone, filtered Gaussian white noise currents indeed induced reliably-timed spike trains (see Fig. 7Aii, top, for twenty overlaid traces, and Fig. 7Aiii, top, for corresponding spike rasters). When Halo and ChR2 were concurrently driven by a Poisson train of yellow and blue light pulses, the spike timings were different from those of spikes elicited in darkness, but were still reliable from trial to trial (see Fig. 7Aii, bottom, and Fig. 7Aiii, bottom, for overlaid traces and spike rasters respectively). Inspection of spike histograms (Fig. 7Aiv) clearly shows that relative to the spike train elicited by current injection in darkness, optically driving Halo and ChR2 could sometimes abolish previously reliable spikes, create new spikes, or advance or delay the timing of specific spikes. We compared mean spike rates for neurons receiving filtered Gaussian white noise current injection alone vs. with additional illumination, and found no difference in spike rates for these two conditions (*p*>0.90, t-test; *n* = 7 neurons; Fig. 7B), indicating that our optical intervention preserved spike rate. However, precise spike timing was altered significantly: cross-correlations of Gaussian current injections delivered alone vs. delivered with concurrent illumination resulted in zero-lag cross-correlations that were on average 37% smaller than cross-correlations of pairs of spike traces resulting from Gaussian current injections alone (*p*<0.005, see Methods for details of this analysis). This indicates that precise spike timing was indeed disrupted by the activation of Halo and ChR2, even while spike rate was preserved. Thus, Halo and ChR2 are synergistic reagents, together enabling two-color controlled excitation and inhibition of individual, genetically-specified neurons. The ChR2/Halo system constitutes an easy-to-use, yet powerful, optical toolbox for the analysis and engineering of genetically-specified circuit elements in the brain, and also opens up the bidirectional optical voltage control of a variety of electrically excitable cells.

## Discussion

We here reveal a genetically-encoded reagent for the safe and effective silencing of neural activity, in the mammalian codon-optimized gene for *N. pharaonis* halorhodopsin (abbreviated Halo). Even a brief pulse of yellow light could completely silence the spiking of a Halo-expressing neuron, and yet allow normal spiking activity within milliseconds after light cessation. Furthermore, individual neurons could express the yellow light-activated chloride pump Halo and the blue-light activated cation channel ChR2, and respond to yellow vs. blue light with oppositely-directed voltage changes. This ability to bidirectionally control neural activity allowed us to disrupt precise spike timing of neural activity without altering spike rate, a long-desired tool for systems neuroscientists interested in the role of synchronous or precisely timed neural activity in the brain. The ability to hyperpolarize and depolarize a neuron at will with multiple colors of light powerfully extends the optical toolbox accessible to the modern neuroscientist. When compared to the pioneering study on optical inactivation of neuronal activity, which used rat rhodopsin 4 (RO4) to enable light-driven G-protein-mediated activation of potassium conductances and inhibition of calcium channels [27], Halo possesses a number of useful properties, perhaps consistent with its being a monolithic protein capable of both sensing light and directly influencing neuronal voltage. The timescales of induction of and release from voltage inhibition by Halo are about 5 and 90 times faster, respectively, than demonstrated for RO4. In addition, Halo mediates light-driven hyperpolarizations of amplitude>350% of those mediated by RO4 or other G-protein mediated methods such as baclofen administration, which may aid in the observed high performance of Halo in neuronal spike blockade, a highly nonlinear process.

Even by itself, Halo presents a number of technological opportunities for the practicing neuroscientist. Although simulating excitation is possible with multiple tools such as ChR2 and glutamate uncaging, in which pulses of UV light cause chemical release of excitatory neurotransmitter [46]–[48], no tool for optical inhibition has yet become widespread. Here we show that even millisecond pulses of light can induce hyperpolarizations of several millivolts, and therefore may be useful in a variety of contexts for simulating background or well-timed inhibitory synaptic activity. Creating fusion proteins in which Halo is targeted to specific locations where inhibitory synapses uniquely cluster–for example, the axon initial segment of certain cell types [49], [50], or other locations where GABAergic receptors accumulate [51]–[53]–may enhance the use of Halo for the study of the function of not only specific cell types, but specific classes of inhibitory synapse.

The ability to functionally lesion brain regions, or cell types, in a rapidly reversible fashion opens up a large class of experiments in which specific neuron populations must be inactivated for precise, sub-second durations during a task. As has been shown in multiple earlier studies, the mammalian brain contains sufficient levels of all-trans retinal *in vivo* to enable function of type I opsins such as ChR2 [28], [29], and so Halo will likely immediately find powerful uses in the intact mammalian brain, and in brain slice experiments. Laboratories working on classical neural model organisms such as *Drosophila* and *C. elegans* have devised ways of delivering all-trans-retinal to the nervous systems of such animals in order to enable ChR2 function [54], [55], and these protocols would therefore also enable the function of Halo in these invertebrates. Accordingly, we predict that the ability to optically silence neural activity in multiple kinds of organism using Halo will transpire in quite short order. In addition, the newly-enabled ability to drive excitation and inhibition of genetically-targeted neurons with blue and yellow light will likely be particularly valuable for probing the role of specific subsets of neurons in neural computations. For example, the ability to drive a specific set of neurons, while inhibiting activity in a subgroup of the neurons that the first set synapses upon, may allow new ways of analyzing the necessity and sufficiency of neural activity in complex, distributed neural circuits. In addition, the ability to play back balanced, yet random, patterns of excitation and inhibition may open up new horizons into understanding the causal role of precise spike timing or neural synchrony in brain function and disease, an area of longstanding yet growing interest.

Optical methods for altering neural circuit function have appeal in part because they can in principle piggyback off of the large amount of effort scientists have invested in microscopy technology development, especially in recent years towards *in vivo* brain imaging. For example, the ability to use optical fibers to image deep neural circuits [56] may enable the ability to stimulate or inhibit specific neurons within deep brain structures. For creation of narrowband, high-intensity light, LEDs and solid-state diode lasers have become progressively inexpensive, bright, and easy-to-use. Two-photon excitation methods will likely prove valuable for driving excitatory and inhibitory opsin activity in neurons up to 1 mm deep [57]. Another key aspect of optical methods of neural control is the speed with which activation and inactivation can take place, since it is trivial to modulate light intensity at high speeds, faster than practically all physiologically relevant processes. Accordingly, in addition to reagents for neural excitation and inhibition, other light-sensitive reagents may prove useful in the endeavor to analyze and control neural circuits. One recent publication revealed how to simulate neurotransmitter receptor activation in response to light, for example [58]. In addition, a few recent papers describe reagents that enable signaling pathways to be driven in response to light, including the creation of the second messenger cyclic AMP (cAMP) in response to light [59], or optical control of the beta-adrenergic signaling pathway using a beta-adrenergic receptor/rhodopsin hybrid protein [60]. Such tools may be useful for testing the causal role of signaling and gene expression changes in specific cells in memory circuits and reward pathways. Nevertheless, non-optical and chemical approaches will continue to find many uses for reliable, enduring inhibition of specific brain circuits and cell types–especially when large regions of deep brain tissue are involved.

From a neuroengineering standpoint, optical prosthetics capable of inhibiting neural activity may present less-invasive strategies for treating disorders of neural hyperactivity. Aside from the very prominent use of pharmacological inhibitors of neural excitability in treating neurological and psychiatric disorders, few non-ablative methods of inhibiting neurons have been described. ChR2 has already proven to be well-tolerated in intact mammalian neural circuits–up to a year, the longest time yet described in a publication [29]; if Halo gains a similar track record, it is possible that Halo-enabled prosthetics may open up new horizons in controlling disorders of excitable cells such as epilepsy [61], depression [12], neuropathic pain [62], and cardiac hyperexcitability [63], [64]. Already the targeting of ChR2 to retinal ganglion cells has been shown to strikingly restore light-driven responses in the visual cortex of mice lacking photoreceptors [29]; in principle, a photoreceptor-lacking retina differentially expressing ChR2 in “on” ganglion cells and Halo in “off” ganglion cells may more accurately convey images from the outside world to the central nervous system. In the immediate future, the ability to study the effects of well-timed neuron or circuit inactivation in animal models of disease may rapidly reveal new principles for selecting neural circuit targets for treatment of specific disorders. Finally, there are implications of the use of Halo in biotechnological scenarios, such as high-throughput drug screening. Several proposals (and even commercially available systems) exist for using electrical stimulation to depolarize excitable cells, thus facilitating the screening of depolarization-activated ion channels (e.g., [65]). However, no comparable method has been proposed for the finding of drugs that target hyperpolarization-activated channels, such as the family of channels mediating the hyperpolarization-activated cation currents I(h) and I(f). Such channels may be useful drug targets for tackling problems such as absence seizures, bradycardia, and other disorders [66]–[69]. An all-optical method for screening for such drugs, which uses light of one frequency to drive hyperpolarization of a cell line containing Halo and the channel of interest, and then uses light of another frequency to observe fluorescence changes of an ion-sensitive chemical or genetically encoded sensor, could revolutionize the process of finding such drugs. Thus, Halo not only presents a number of unique features that will enable effective, and rapidly inducible and reversible, inhibition to be applied to a number of neural circuit questions, but will likely open up new horizons in biotechnology as well.

## Materials and Methods

### Plasmid constructs

*N. pharaonis* halorhodopsin with mammalian-optimized codon usage (hereafter abbreviated as Halo) was synthesized by Epoch Biolabs (Sugar Land, Texas, USA). (876 base pairs, DNA sequence ATGACTGAGACCCTCCCACCCGTGACTGAAAGCGCCGTCGCTCTGCAAGCAGAGGTTACCCAGCGGGAGCTGTTCGAGTTCGTCCTCAACGACCCCCTCCTGGCTTCTAGCCTCTACATCAACATTGCTCTGGCAGGCCTGTCTATACTGCTGTTCGTCTTCATGACCAGGGGACTCGATGACCCTAGGGCTAAACTGATTGCAGTGAGCACAATTCTGGTTCCCGTGGTCTCTATCGCTTCCTACACTGGGCTGGCATCTGGTCTCACAATCAGTGTCCTGGAAATGCCAGCTGGCCACTTTGCCGAAGGGAGTTCTGTCATGCTGGGAGGCGAAGAGGTCGATGGGGTTGTCACAATGTGGGGTCGCTACCTCACCTGGGCTCTCAGTACCCCCATGATCCTGCTGGCACTCGGACTCCTGGCCGGAAGTAACGCCACCAAACTCTTCACTGCTATTACATTCGATATCGCCATGTGCGTGACCGGGCTCGCAGCTGCCCTCACCACCAGCAGCCATCTGATGAGATGGTTTTGGTATGCCATCTCTTGTGCCTGCTTTCTGGTGGTGCTGTATATCCTGCTGGTGGAGTGGGCTCAGGATGCCAAGGCTGCAGGGACAGCCGACATGTTTAATACACTGAAGCTGCTCACTGTGGTGATGTGGCTGGGTTACCCTATCGTTTGGGCACTCGGCGTGGAGGGAATCGCAGTTCTGCCTGTTGGTGTGACAAGCTGGGGCTACTCCTTCCTGGACATTGTGGCCAAGTATATTTTTGCCTTTCTGCTGCTGAATTATCTGACTTCCAATGAGTCCGTGGTGTCCGGCTCCATACTGGACGTGCCATCCGCCAGCGGCACACCTGCCGATGACTGA). The Halo-GFP fusion protein sequence was generated by PCR amplification of the Halo gene with primers 5′-GAATTCGCCACCATGACTGAGACCCTCCCACCCGTG and 3′-GGATCCGTCATCGGCAGGTGTGCCGCTGGC and inserted into the EcoRI and BamHI sites of pEGFP-N3 (Clontech), which has the CMV promoter. The Halo-GFP fusion protein sequence was then PCR amplified with primers 5′-CCGGTGCCACCATGACTGAGACCCTCCCACCCGTG and 3′-GAATTCTTACTTGTACAGCTCGTCCATGCC and inserted into the lentiviral vector FCK(1.3)GW containing the CaMKII promoter [35] via AgeI and EcoRI sites, to make vector FCK-HaloGFP. All constructs were verified by thorough sequencing. The channelrhodopsin construct used in this paper, FCK-hCmC, contains the human codon-optimized gene ChR2 (sequence found at [70]) fused to the fluorescent protein mCherry [38], under the CaMKII promoter [35].

### Hippocampal neuron culture and imaging

Sprague-Dawley rats (Charles River) were handled in accordance with the guidelines of the MIT Committee on Animal Care. Hippocampal regions CA3-CA1 of postnatal day 0 or day 1 rats were isolated and treated with trypsin (1 mg/ml) for 12 min. Digestion was stopped by Hanks solution supplemented with 20% fetal bovine serum and trypsin inhibitor. Tissue was dissociated with silicone-coated Pasteur pipettes and centrifuged at 1000 rpm at 4°C for 10 min. Dissociated neurons were plated on glass coverslips pre-coated with Matrigel (BD Biosciences) at a rough density of approximately two hippocampi per 24 coverslips. Neurons were transfected using a commercially available calcium phosphate transfection kit (Invitrogen), at 3–5 days in vitro. GFP fluorescence was used to identify successfully-transfected neurons, indicating a net transfection efficiency of ∼7%. All images and electrophysiological recordings were made on 9–15 day -in-vitro neurons (approximately 6–10 days after transfection). Confocal images of transfected neurons were taken with a Zeiss LSM 510 confocal microscope. Cell death count was carried out on living cultures, seven days after transfection, by adding 4 µM ethidium homodimer-1 (Invitrogen) to the culture medium for 10 mins at 37°C, then washing the cells with Tyrode's solution (see below). GFP-positive and negative neurons were counted for positive and negative ethidium fluorescence, in three regions on each of five coverslips, for this viability assay.

### Electrophysiology and optical methods

Whole cell patch clamp recording was made on 9–15 day-in-vitro neurons using a Multiclamp 700B amplifier, connected to a Digidata 1440 digitizer (Molecular Devices) attached to a PC running pClamp 10. During recording, neurons were bathed in Tyrode's solution containing (in mM) 150 NaCl, 2.4 KCl, 2 CaCl, 2 MgCl, 10 HEPES, 10 Glucose, 10 µM NBQX, 10 µM gabazine and 50μM D-APV. Borosilicate glass (Warner) pipettes were filled with a solution containing (in mM) 130 K-Gluconate, 7 KCl, 2 NaCl, 1 MgCl2, 0.4 EGTA, 10 HEPES, 2 ATP-Mg, 0.3 GTP-Tris and 20 sucrose. Pipette resistance was ∼5 MΩ and the access resistance was 10–25 MΩ, which was monitored throughout the voltage-clamp recording. Resting membrane potential was −52 to −70 mV in current-clamp recording.

Photocurrents were first measured with pairs of 1-second long light pulses, separated by periods of darkness lasting 1 second, while holding neurons in voltage clamp at −70 mV, −30 mV and+10 mV to assay the properties of Halo. Light-induced membrane hyperpolarizations were induced by 1 s duration light pulses, separated by periods of 1 s in the dark, in neurons current-clamped at resting membrane potential. Light pulse trains were synthesized by custom software written in MATLAB (Mathworks), then played to the DG-4 light source through a digital-to-analog converter on the Digidata 1440. For the spike-blockade experiment, spikes were first induced via somatic current injection through the patch pipette; neurons easily fired repeated action potentials with ∼100% probability, in response to ∼300 pA current injections (4 ms duration).

A DG-4 optical switch with 300-W xenon lamp (Sutter Instruments) was used to deliver all light pulses, for Halo or ChR2 activation. A Texas Red filter set (Chroma, excitation bandpass 560±27.5 nm, dichroic 595LP) was used to deliver yellow light to activate Halo. The same dichroic mirror was also used to deliver blue light, but with a standard GFP excitation filter (bandpass 480±20 nm) in the DG-4, to allow ChR2 excitation. Note that the 595LP dichroic mirror only reflects 35% of 460–500 nm light through the objective; custom-coated dichroics that reflect light all the way into the ultraviolet (as are available from companies such as Chroma) would be optimal.

Precise timing-disruption experiments were conducted by using stimuli modeled after those in [45]. Briefly, we current-clamped cultured hippocampal neurons with a 4-second long Gaussian white noise current (mean 40–100 pA, standard deviation 20–70 pA) low-pass filtered with a first-order Butterworth filter with cutoff frequency of 100 Hz. Concurrently, neurons were, on half the trials, also exposed to a mean inter-pulse interval λ = 100 ms Poisson train of alternating yellow and blue light pulses. Identically-shaped, filtered Gaussian white noise currents, and the same optical Poisson train, were used for all trials on all neurons to facilitate comparison. For each neuron, we acquired between 10 and 20 traces in each of the dark and illuminated conditions, taking equal numbers in each condition to facilitate data analysis.

### Data analysis

Amplitude and timing data were analyzed using Clampfit 10 (Molecular Devices) and custom analysis scripts written in MATLAB. Onset times and offset times of currents and voltages were measured as the duration the electrical signal takes to transition between 15% and 85% of peak value, with respect to baseline. Statistics analysis was done with Prism (GraphPad Software Inc.) and Statview (SAS Institute). Figure 6A was created using DigitizeIt software applied to the cited source materials (ShareIt Inc., Germany).

Precise spike timing experiments were analyzed using Clampfit 10 and MATLAB. Specifically, the rasters in Fig. 7Aiii were made by thresholding voltage traces at −10 mV in Clampfit, which cleanly isolated spikes from non-spike membrane fluctuations. The spike time histogram in Fig. 7Aiv has a bin size of 500 µs. The cross-correlation analysis (Fig. 7C) was performed by first assigning each spike a 1 ms long presence (1 ms of ones) centered around the spike peak, and assigning the rest of the trace zeros. Then, we computed cross-correlations for each neuron between spike trains elicited from the same filtered Gaussian white noise current injection, played twice, when either both current injections were performed in the dark (black trace in Fig. 7C), or when one of the current injections was performed while a Poisson train of yellow and blue light pulses was concurrently delivered (green trace in Fig. 7C). For each neuron, cross-correlations were first computed between all possible pairs within a pairing condition (i.e., dark vs. dark, and dark vs. light). Individual traces were never cross-correlated with themselves, for obvious reasons. Then, for each of the two pairing conditions, the cross-correlation curves were averaged across all pairs in the pairing condition. Finally, for each neuron, each of these two averaged curves was then normalized to the maximum value taken by either averaged curve, to facilitate comparison of the effect of optical modulation on precise spike timing across different neurons.
